# A Gentleman with a Grievance

**Published:** 1887-11-12

**Authors:** 


### The Hospital

A Weekly Institutional Journal of

### Science, Medicine, and Philanthropy.

*For Hospitals, Asylums, Medical Practitioners, Students, Nurses, Families, Parishes, Congregations, and the Charitable Public.*

Vol. III. No. 59] [November 12, 1887.

### "A GENTLEMAN WITH A GRIEVANCE."

There is no reason in the world why hospitals should not be attacked with the weapons of criticism; there is every reason why, if they are so attacked, the onslaught should be conducted fairly. To a grown-up man a tin sword may not be a very alarming instrument, though to a child it may suggest Bluebeards, murderous Turks, guillotines, and nobody knows what. Such a weapon was flourished in the *Pall Mall Gazette* a few days ago, and if the public generally were as well informed about hospitals as they ought to be, the valiant De la Mancha, its owner, might have been allowed to disport himself undisturbed so long as it afforded him satisfaction to do so. Unhappily, the public is very ignorant about hospital regulations and defences, and is inclined to believe that a very feeble attack of criticism is sufficient to inflict upon them irreparable damage. They see the attack, and if no defence is made, they imagine it is because no defence is possible.

The writer in the *Pall Mall* has "exploited," as he expresses it, two hospitals, whose identity he seeks to disguise by two terms dug up from among the fossil remains of extinct theological controversies —terms which suggest an elementary acquaintance with the earlier chapters of Kurtz. From this it may be surmised that he belongs either to the genus cleric, or to that very considerable body of persons whom somebody has described as "ecclesiastically-minded laymen." Whether, however, "cleric" or "laic" be his proper designation, he confesses to a circumstance with which, in either event, there can have been no difficulty in becoming familiar. He admits the soft impeachment of limited resources—so limited as to prevent him carrying himself and his "Bardolphian nose," as he calls it, to the private consulting-rooms of a certain eminent specialist. A "doctor" he apparently had, who failed to cure his nose; a failure which seems to have excited no surprise in either physician or patient. The family Esculapius being worsted in the conflict, Bardolph bethinks himself of a more redoubtable champion in the field of physic. But here his difficulties begin. The eminent one is not merely a specialist in practice, but also in the matter of fees.

When the family doctor is sounded on the delicate subject of the transference of the "Bardolphian nose" and its owner from his care to that of his more famous brother in the out-patient department, he vetoes the suggestion at once; not, be it observed, that he objects to losing a patient, but because he is of opinion that "Bardolph" is not a fit and proper person for an out-patient; and that his unfitness will be only too evident to the specialist and any other watchdog who may be set to defend the hospital from hungry wolves. The venture was, however, made under the disguise of an "unemployed" hat and a Trafalgar Square *tenue*. "Bardolph" thus arrayed succeeded in deceiving the authorities at one of the hospitals he had elected to "exploit." By arrangement with the secretary, who seems to have suspected the possibility of a larger fee being available, he pays his shilling and takes his turn among the out-patients. He is examined, diagnosed, prescribed for, and dismissed in three minutes, notwithstanding that he is very anxious to explain certain symptoms which he thinks have not been sufficiently taken into account.

That we gather is his first grievance. The eminent specialist did not give him time enough. Now we have to ask, How does he know that there was not time enough given? According to his own showing the affection was a "surface" one, and probably of a kind perfectly familiar to the specialist. It seems even to have been well known to the assembled students, because the remarks made were all of a character appropriate to a typical example of a well-known disease. In many cases of skin disease a period of three minutes is ample for all practical purposes when the physician is master of his business. On that point therefore there was absolutely no ground of complaint whatever. It must be remembered that large numbers of out-patients are afflicted with very trifling ailments, disorders of digestion, and the like. For many of these cases, as for certain skin affections, which merely to see is to diagnose and to know how to treat, two or three minutes are all the doctor requires, and all that would be of the least service to the patient. There are undoubtedly patients in the out-patient department which require more than three minutes, and we venture to affirm that such, as a rule, get all the time needed for a careful diagnosis, whether three or thirteen or thirty minutes be required. Of course among so many thousands there must be instances where occasional injustice is done; but surely that is only saying that physicians and students are human, and that hospitals are human institutions. Did "Bardolph" never meet with a perfunctory clergyman, or a careless schoolmaster, or a foolish newspaper writer?

The next scene in the play—for we do not take the critic seriously—is hardly what might have been expected from a highly moral Daniel come to judgment. After the examination was over two bottles were given to the patient; one of them containing about a quart of lotion, the other a similar quantity of physic. What did this virtuous gentleman do with these bottles? He "deposited them both in a corner of the first quiet street he got to." After this confession that he never gave either medicine or lotion a single trial, he logically and unblushingly sums up the results of his experiment thus: "My first experience was a failure then." Can any conclusion be more inconsequent, not to say impudent? If he had been a boy instead of a man, the proper reward for such utterly frivolous behaviour would have been a sound boxing of the ears.

At hospital number two the out-patients' waiting-rooms are described as damp and draughty vaults. That is possibly the case; and no doubt the hospital authorities if they had means and space at disposal would be exceedingly glad to dispense with both vaults and draughts. For let it not be forgotten that if patients have to go into inconvenient and unpleasant places, so have hospital officials and doctors. And if the public desires the improvement of any particular hospital in this respect, it has the remedy entirely in its own hands. Let it but supply the needful, and the necessary changes will be made without the loss of an hour. In due time the patient saw the second specialist, who "went into ecstasies" over the case, and produced a favourable impression of "care and evident comprehension of the whole thing." Unhappily, when a second and third visit were made, the particular specialist seen before was not present. We now know that he had gone for his holiday. The people, therefore, waited for some time in vain. It is not said that no substitute was in attendance; and, indeed, the public has since been informed, that there was a substitute, and that that was the reason no official announcement was made.

Now we submit that, for a responsible journal to publish a trumpery experience of this kind as a grave indictment against hospitals, is ridiculous. It is specially absurd on the part of the *Pall Mall Gazette*, which prides itself on its absolute fairness and candour. The writer says he pleads guilty to "buttoning up his pockets" with a "strong remark" when young ladies canvass him for donations. That is to say, there are institutions in London which are the pride and glory of Englishmen and the admiration of foreigners all the world over, because of the noble principles on which they are founded and the excellent work they do; and yet because a casual visitor thinks he does not receive sufficient attention at one, and fails to meet with the same doctor at another twice in succession, he must needs button up his pockets against them all, and by implication invite all his readers to do the same. Is this fair criticism, or is it a poor attempt at sensational "copy" making? We yield to no one in our respect for newspaper writers who boldly and plainly speak the truth to the public; but we contend that the habit of plain speaking should always be associated with a broad and liberal judgment, and the most careful regard for facts. Statements should not merely be correct, they should also be wise and just. Let hospitals be criticised by all means, but to refuse subscriptions altogether on such frivolous grounds as these is like condemning a soldier to be shot because he has neglected to pipe-clay three square inches of his belt.

